# Quercetin, a flavonoid, combats rotavirus infection by deactivating rotavirus-induced pro-survival NF-κB pathway

**DOI:** 10.3389/fmicb.2022.951716

**Published:** 2022-08-02

**Authors:** Shreya Banerjee, Rakesh Sarkar, Arpita Mukherjee, Shin-ichi Miyoshi, Kei Kitahara, Prolay Halder, Hemanta Koley, Mamta Chawla-Sarkar

**Affiliations:** ^1^Division of Virology, ICMR-National Institute of Cholera and Enteric Diseases, Kolkata, West Bengal, India; ^2^Division of Pharmaceutical Sciences, Graduate School of Medicine, Dentistry and Pharmaceutical Sciences, Okayama University, Okayama, Japan; ^3^Collaborative Research Center of Okayama University for Infectious Diseases in India, Okayama University, ICMR-National Institute of Cholera and Enteric Diseases, Kolkata, West Bengal, India; ^4^Division of Bacteriology, ICMR-National Institute of Cholera and Enteric Diseases, Kolkata, West Bengal, India

**Keywords:** rotavirus, quercetin, NF-κB, antiviral therapeutics, phytochemical

## Abstract

Rotavirus (RV) is the leading cause of acute gastroenteritis and watery diarrhea in children under 5 years accounting for high morbidity and mortality in countries with poor socioeconomic status. Although vaccination against RV has been implemented in more than 100 countries, the efficacy of vaccine has been challenged in low-income settings. The lack of any FDA-approved drug against RV is an additional concern regarding the treatment associated with rotavirus-induced infantile death. With the purpose for the discovery of anti-RV therapeutics, we assessed anti-rotaviral potential of quercetin, a well-characterized antioxidant flavonoid. *In vitro* study revealed that quercetin treatment resulted in diminished production of RV-SA11 (simian strain) viral particles in a concentration-dependent manner as estimated by the plaque assay. Consistent with this result, Western blot analysis also revealed reduced synthesis of viral protein in quercetin-treated RV-SA11-infected MA104 cells compared to vehicle (DMSO) treated controls. Not surprisingly, infection of other RV strains A5-13 (bovine strain) and Wa (Human strain) was also found to be abridged in the presence of quercetin compared to DMSO. The IC_50_ of quercetin against three RV strains ranges between 2.79 and 4.36 Mm, and S.I. index is greater than 45. Concurrent to the *in vitro* results, *in vivo* study in mice model also demonstrated reduced expression of viral proteins and viral titer in the small intestine of quercetin-treated infected mice compared to vehicle-treated infected mice. Furthermore, the result suggested anti-rotaviral activity of quercetin to be interferon-independent. Mechanistic study revealed that the antiviral action of quercetin is co-related with the inhibition of RV-induced early activation of NF-κB pathway. Overall, this study delineates the strong anti-RV potential of quercetin and also proposes it as future therapeutics against rotaviral diarrhea.

## Introduction

Rotavirus (RV) infection causes severe gastroenteritis among infants below 5 years of age, accounting for 128500 deaths globally in the year 2016, with the highest incidence in Sub-Saharan Africa, Southeast Asia, and South Asia (Clark et al., 2017; Crawford et al., 2017; Troeger et al., 2018). Inaugurating RV vaccination program in universal immunization program (UIP) has decreased the burden of rotaviral diarrhea-specific mortality by 25% and hospitalization by 40% in the past decade globally (Curns et al., 2010; Jiang et al., 2010; Bayard et al., 2012; Yen et al., 2014; Abou-Nader et al., 2018; Burnett et al., 2018, 2020; Aliabadi et al., 2019). Nevertheless, the efficacy of the RV vaccines differs greatly depending on the socioeconomic situation of the country. In low/middle-income countries (LMIC) with higher endemicity, chances of intergenotypic reassortments are higher due to co-circulation of multiple strains, enteric co-infection, zoonotic transmission, and genetic drift of neutralizing antigen (Matthijnssens et al., 2009; Martella et al., 2010; Bányai et al., 2012; Banerjee et al., 2018; Clark et al., 2019). Along with these factors, vaccine immunogenicity is also compromised due to other aspects such as co-administration of oral polio vaccines, maternal antibodies, and neutralizing factors present in breast milk and malnutrition (Chen et al., 2017; Saha et al., 2021). When challenged with this unending introduction and accumulation of point mutations leading to viral heterogeneity, only prophylactic vaccination might not be sufficient against rotavirus infection in LMIC.

RV, a non-enveloped double-stranded RNA virus belonging to the family of Reoviridae, comprises of three concentric layers of capsid proteins which encloses at its core 11 segmented dsRNA genome. Outermost layer consists of glycoproteins VP7 and VP4, the middle layer consists of VP6 trimers, and the innermost layer consists of VP2 trimers. The genome codes for six structural viral proteins (VP1, VP2, VP3, VP4, VP6, and VP7) and six non-structural proteins (NSP1, NSP2, NSP3, NSP4, NSP5, and NSP6). Rotavirus primarily infects enterocytes leading to malabsorption as well as diarrhea stimulated by rotavirus enterotoxin NSP4 (Silvestri et al., 2004; Greenberg and Estes, 2009; Desselberger, 2014; Crawford et al., 2017). Upon infection, rotavirus triggers a plethora of host cellular signaling pathways which is essential for their propagation and abrogates certain cellular defense machinery for their own ease (Chemello et al., 2002; Dutta et al., 2009; Bagchi et al., 2010; Crawford et al., 2012; Eichwald et al., 2012; Bhowmick et al., 2013; Chattopadhyay et al., 2013, 2017; Chanda et al., 2015, 2016; Ding et al., 2018; Mukherjee et al., 2018; Sarkar et al., 2020; Patra et al., 2021).

Quercetin (C15H10O7, 3,5,7,3′,4′-pentahydroxyflavone) is a flavonoid found in a variety of fruits, vegetables, leaves, seeds, and grains, including apples, berries, brassica vegetables, capers, grapes, onions, spring onions, tea, and tomatoes. The antioxidant, anti-inflammatory, and pro-apoptotic effects of the highly bioactive compound quercetin have been well documented (Li et al., 2016; Salehi et al., 2020). Various reports established the fact that quercetin plays vital role in regulating metabolism, inhibiting cardiovascular diseases, and preventing cancer with anti-proliferative and growth-suppressing effects along with other cellular functions (Russo et al., 2012). Besides other diseases, quercetin is reported to exert antiviral effects on few viruses like influenza, dengue, Ebola, and rhinovirus (Ganesan et al., 2012; Wu et al., 2015; David et al., 2016; Dewi et al., 2020; Fanunza et al., 2020; Mehrbod et al., 2020). Quercetin is found to interact with influenza hemagglutinin protein subsequently inhibiting viral-cell fusion (Wu et al., 2015). Ebola virus infection is attenuated by quercetin which specifically targets anti-IFN activity of EBOV VP24 (Fanunza et al., 2020). Quercetin is also reported to hinder rhinovirus replication, endocytosis, and viral protein synthesis (Ganesan et al., 2012). A study on antiviral effect of few phytochemicals on rotavirus infection also showed poor anti-rotaviral effect of quercetin compared to rhamnoglycosides hesperidin and poncirin, though no mechanism was studied (Bae et al., 2000).

A pilot study was performed to assess the anti-rotaviral effects of various phytochemicals and their mechanism of action. Compared to other phytochemicals which were tested such as epigallocatechin, fisetin, curcumin, 6-gingerol, baicalin, and quercetin, we observed very strong antiviral effect of quercetin at lowest, non-toxic doses *in vitro*. Thus, a further in-depth study was conducted to analyze the anti-rotaviral mechanism of quercetin.

Our primary observation revealed significant reduction in total viral protein synthesis, viroplasm formation, and infectious virus production in cells (*in vitro*) and suckling mice (*in vivo*) treated with quercetin. Subsequent mechanistic studies revealed quercetin hinders RV replication by subduing RV-induced activation of pro-viral NF-κB pathway. Overall, this study delineates an excellent therapeutic potential of the plant-based flavonoid quercetin against rotavirus infection *in vitro* as well as *in vivo via* modulating activation of pro-survival NF-κB pathway.

## Materials and methods

### Cell culture and *in vitro* rotavirus infection

Monkey kidney cell line MA104 (ATCC number: CRL-2378^™^) was cultured in Minimal Essential Medium (MEM) supplemented with 10% fetal bovine serum (FBS) and 1% antibiotic-antimycotic solution (Invitrogen, Carlsbad, CA, USA) and maintained in a 5% CO_2_ at 37°C humidified incubator. Four cell culture-adapted rotavirus strains: simian strain SA11, human strain Wa, bovine wild-type strain A5-13, and bovine NSP1-mutant A5-16, were used for the *in vitro* study. RV strains were activated with acetylated trypsin for 1 h at 37°C prior to infection. MA104 cells were infected with activated RV strains at three multiplicities of infection (MOI) as described previously (Dutta et al., 2009). For all experiments, the time of virus addition was considered as 0 h post-infection (Chattopadhyay et al., 2013). In dose-dependent response study, quercetin was added to the media at 1 h post-infection.

### Rotavirus infection in mice model

BALB/c mice were raised as per institutional protocol in filter-topped cages on a standard rodent diet with water available *ad libitum*. The experiments were executed conferring to national regulations and approved by the concerned animal ethics committee. Five-day-old mice were inoculated with RV-EW (10^7^ PFU) and RV-SA11 (10^7^ PFU) through oral gavages. After 12 h of virus inoculation, mice were treated with either quercetin (10 mg/kg/day) or equivalent amount of DMSO solution for 1 and 2 days by oral gavages. After 1 and 2 days of treatment, mice were euthanized to collect intestine, and small intestinal homogenate was prepared. Subsequently, viral protein (VP6) expression from small intestinal homogenate of DMSO or quercetin-treated mice was determined by Western blot analysis. Stool samples were collected from both treated and untreated mice after 48 h of treatment, and estimation of number of viral particles present in the stool was done by using Premier® Rotaclone® (Meridian Bioscience, Inc.) according to the manufacturer's instructions. Small intestine of suckling mice was isolated and stored in 4% (v/v) formalin prior to HE staining.

### Reagents and antibodies

Quercetin (Q4951) was obtained from Merck (Sigma-Aldrich, USA) and dissolved in DMSO to prepare stock solution of 100 mM. MEM (Gibco, 41500-067) and FBS (Gibco, 10270-106) were purchased from Thermo Fisher Scientific, USA. Protease inhibitor cocktail (P2714), phosphatase inhibitor cocktail 2 (P5726), MTT (M5655), TNFα (H8916), and Bay11-7082 (B5556) were procured from Merck (Sigma-Aldrich, USA).

Rabbit polyclonal antibody against phospho-NF-κB p65 (sc-101752), anti-β-actin HRP-conjugated (sc-47778 HRP), and mouse monoclonal antibodies against RV-VP6 (sc-101363) were purchased from Santa Cruz Biotechnology (USA). Rabbit polyclonal antibodies against NF-κB p65 (#3034), NF-κB1 (p-105/p-50) (#3035s), phospho-IκBα (Ser32) (#9241s), IκBα (#9242), p-IKKα/β (#2697), Rel-B (#4954s), phospho-JAK1 (#3331s), and JAK1 (#3332s) were obtained from Cell Signaling Technology, USA. Mouse monoclonal antibody against p-STAT1 (#612132), STAT1 (#610185), and IKKβ (#611254) was purchased from BD Biosciences, USA. Antisera against RV-SA11 structural and non-structural proteins were raised either against peptide (for NSP1) or by purifying full-length protein synthesized in bacterial expression system (for VP1) in rabbits following the standard protocols of the Department of Virology and Parasitology, Fujita Health University School of Medicine, Aichi, Japan.

### Western blot

Cells were scraped and washed with ice-cold PBS followed by lysis by dissolving in RIPA buffer (Bhowmick et al., 2015). The protein concentration of the cell lysate was measured by Pierce^™^ BCA Protein Assay Kit (Thermo Scientific^™^). The measured cell lysate was boiled for 10 mins after mixing with protein sample buffer (Bhowmick et al., 2015) and further subjected to run for SDS-PAGE. Next, immunoblotting was with specific antibodies as described previously (Bagchi et al., 2010). Primary antibodies were detected using HRP-conjugated secondary antibody (Thermo Scientific^™^) and chemiluminescent substrate (Millipore & Bio-Rad) within ChemiDoc Imaging System (Bio-Rad). All experiments were done in triplicate. The immunoblots shown here represent one experiment from three independent experiments. Relative fold changes in viral protein expression shown in the figure were measured after normalizing against β-actin using Image Lab software (version 5.2.1), BioRad.

### Immunofluorescence confocal microscopy

To perform immunofluorescence, MA104 cells were grown on coverslips placed in 35 mm plates. 60% confluent cells are infected with rotavirus strains followed by treatment with either quercetin or DMSO and processed as described previously (Mukherjee et al., 2018). Primary staining was done with anti-NSP5 antibody raised in rabbit followed by secondary staining with DyLight488-labeled goat anti-rabbit antibody (Thermo Scientific^™^). Cells were stained and mounted with 4′, 6′-diamidino-2-phenylindole (DAPI) (F6057) from Merck (Sigma-Aldrich, USA). Lastly, examination and imaging were done in Zeiss Axioplan microscope (63× oil immersion). Percentage positivity of viroplasm-containing cells was calculated by analyzing 150 cells (50 cells from each of three experimental replicates) by using the formula (Number of cells with punctate viroplasms/Total number of DAPI positive cells) × 100 (Patra et al., 2019).

### Transfection of pcD-NSP1

Full-length NSP1 cloned in pcDNA6 (Invitrogen) (Bagchi et al., 2010) and empty pcDNA6 vector (control) was transfected in MA104 cells using Lipofectamine 2000 reagent (Invitrogen) according to manufacturer's instructions.

### Cell viability assay

Cytotoxicity of quercetin on MA104 cells was examined by cell viability assay conducted in 96-well plates at 80–90% cell confluency. Cells were treated with quercetin at indicated concentrations for 48 h followed by MTT assay. Briefly, 10 μl of MTT solution (5 mg/ml in PBS) was added and incubated at 37°C for 4 h. The formazan complex was dissolved in 200 μl MTT solvent (4 mM HCl, 0.1% Nonidet P-40 in isopropanol) and the optical density (OD) of the solutions was measured at 570 nm. Percentage of cell viability was measured by the formula (OD_Sample_-OD_Blank_) × 100/(OD_Control_-OD_Blank_) (Patra et al., 2019).

### Determination of IC_50_, CC_50_, and IC_50_

50% cytotoxic concentration (CC_50_) and 50% inhibitory concentration (IC_50_) of quercetin were calculated using GraphPad Prism (Version 5) software using non-linear regression model [Y = Bottom + (Top-Bottom)/{1 + 10ˆ[(LogIC_50_-X)^*^Hillslope]}] (Patra et al., 2019). Selectivity index (SI) was derived by dividing CC_50_ with IC_50_ (SI = CC_50_/IC_50_). The estimated CC_50_, IC_50_, and SI of quercetin against different rotavirus strains (SA-11, A5-13, Wa) are presented in Table 1.

**Table 1:** IC_50_, CC_50_, and SI of quercetin against three RV strains.

**Virus strain**	**Quercetin**		
	**IC_**50**_ (μM)**	**CC_**50**_ (μM)**	**SI (CC_**50**_/ IC_**50**_)**
RV-SA11	4.36	>200	>45.87
RV-A5-13	2.73	>200	>73.26
RV-Wa	4.05	>200	>49.38

### Plaque assay

Infectious virus particles were assessed by plaque assay which was performed according to formerly described procedures (Sarkar et al., 2021). Viral plaque-forming unit (PFU) was calculated as PFU/ml (of original stock) = (1/dilution factor) × (number of plaques) × 1/(ml of inoculum/plate) (Smith et al., 1979).

### HE staining of small intestine of mice

The small intestine isolated from 5-day-old suckling mice was fixed immersed in 4% (v/v) formalin, dehydrated afterward in sequential order of ethyl alcohol [50, 70, and 90% (v/v)], xylene/ethyl alcohol solution [50 and 90% (v/v)], and repeatedly twice in xylene. Then, the transparent tissue was paraffin-embedded followed by section with 4-μm thickness. After deparaffinating according to the reverse order used for dehydration, hematoxylin–eosin (HE) staining was done for 8 min. Next, through dehydration and mounting, the HE staining section was prepared for observation.

### Statistical analysis

Mean ± standard deviation (SD) of at least three independent experiments (*n* ≥ 3) was considered for analyses. For band intensity analysis, quantification of viral RNA, cell viability assay, viroplasm positivity assay, and virus yield assay, *p* was calculated using unpaired student's t-test and *p* < 0.05 was considered as statistically significant.

## Results

### Quercetin restricts rotavirus protein expression, viroplasm formation, and infectious particle yield *in vitro*

**Figure 1:**
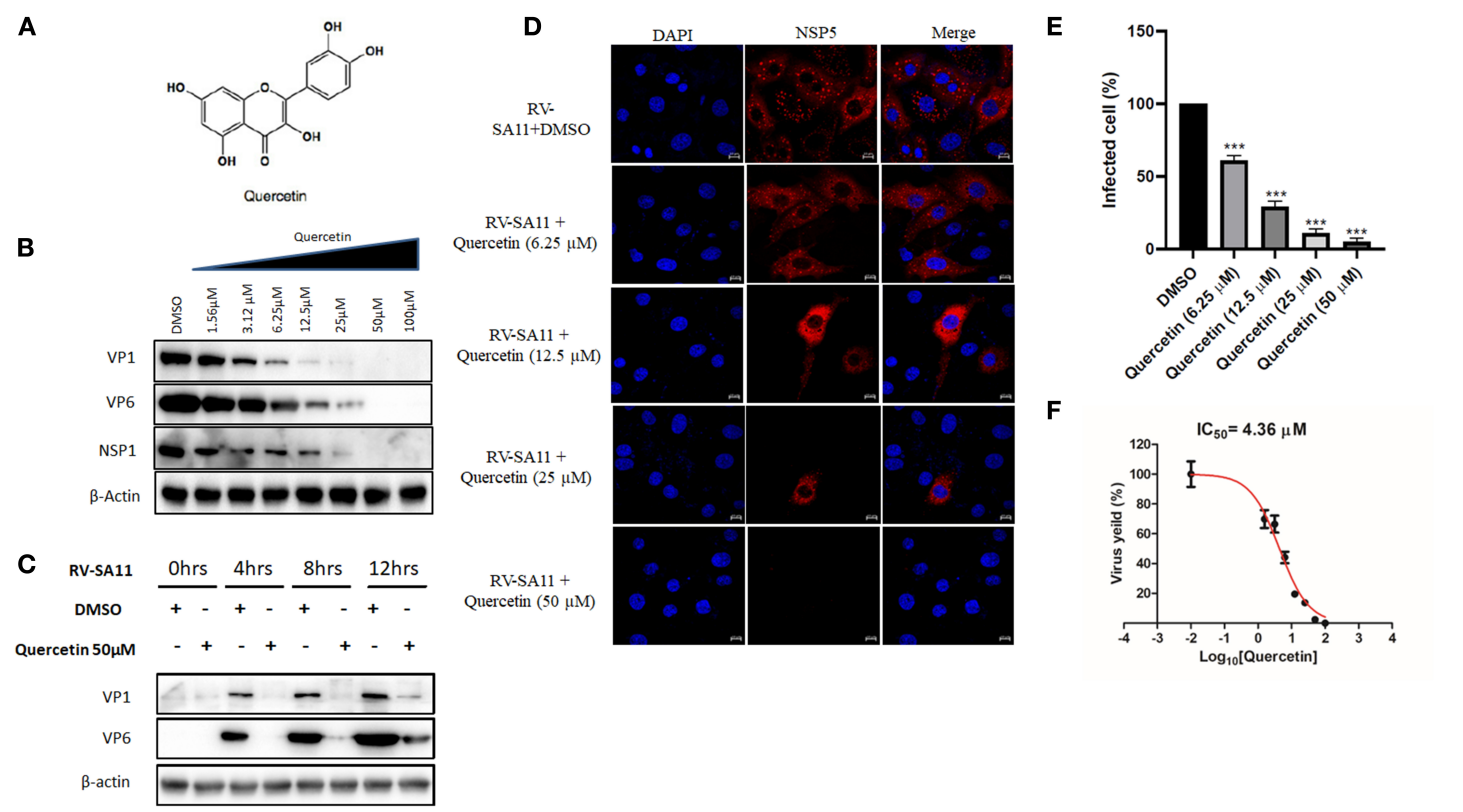
Quercetin treatment diminishes rotavirus replication *in vitro*. **(A)** Chemical structure of quercetin is shown. **(B)** Western blot is performed from SA11(MOI 3)-infected MA104 cells (12 hpi) treated with DMSO or quercetin (1.56, 3.125, 6.25, 12.5, 25, 50, and 100 μM) to evaluate the expression of viral proteins VP1 and VP6. β-actin is taken as internal loading control. **(C)** Protein expression of VP1, VP6, NSP1, and β-actin was checked by immunoblot from SA11-infected MA104 cells treated either DMSO or quercetin (50 μM) incubated for 0, 4, 8, and 12 hpi, respectively. **(D)** Immunofluorescence was performed with RV-SA11 (MOI 3)-infected MA104 cells in the presence of DMSO and quercetin (6.25, 12.5, 25, and 50 μM). At 8 hpi, cells were fixed and permeabilized followed by staining with anti-NSP5 antibody. Secondary staining was done with rhodamine-labeled anti-rabbit antibody. DAPI was used for mounting. Finally, cells were visualized with confocal microscope (63× oil immersion); scale bar 10 μm. **(E)** Minimum of 100 cells from different fields from each experimental slide were selected randomly and analyzed for the quantification of viroplasm-positive cells. The data were represented as mean viroplasm-positive cells (%) ± SD of three experimental replicates. **(F)** Plaque assay was performed from RV-SA11 (MOI 1)-infected MA104 cell treated with graded concentration of quercetin (1.56, 3.125, 6.25, 12.5, 25, 50, and 100 μM) for 24 hpi to measure IC_50._ Each bar represents mean ± SD of three independent experiments (Unpaired student's t-test, ***p < 0.001).

To investigate the dose-dependent response of quercetin (Figure 1A) on RV replication, MA104 cells were infected with RV-SA11 (MOI-3) in the presence of either vehicle control (DMSO) or increasing dose of quercetin (1.56, 3.125, 6.25, 12.5, 25, 50, and 100 μM) and incubated for 12-h post-infection (hpi). Cell lysates were used to quantify the expression of viral proteins using Western blot. The results showed quercetin treatment caused gradual depletion of both RV structural proteins (VP1 and VP6) and non-structural protein (NSP1) in a dose-dependent manner compared to DMSO treatment (Figure 1B). Moreover, in the absence of RV infection, quercetin treatment showed no significant cytotoxic effects on MA104 cells up to 200 μM compared to only DMSO treatment (Supplementary Figure S1). To further assess time-dependent effects of quercetin, lysates are prepared from RV-SA11-infected MA104 cells either treated with solvent DMSO or quercetin (50 μM) post-adsorption and incubated for 0, 4, 8, and 12 hpi, respectively. Immunoblot analysis showed, in the DMSO-treated cells, as RV proteins VP1 and VP6 increase with infection time course, proportional decline in VP1 and VP6 levels is prominent in cells treated with quercetin at those different incubation time points (Figure 1C). To further confirm anti-RV role of quercetin, confocal microscopy was performed to visualize viroplasm-positive cells in response to treatment with quercetin at 8 hpi. Viroplasms were marked with antibody specific for NSP5. Quercetin treatment resulted in gradual reduction in viroplasm-positive cells with increasing concentrations of quercetin treatment (Figures 1D,E). Consistent with this data, plaque assay also showed dose-dependent inhibition of infectious RV particle formation in the presence of quercetin compared to DMSO treatment at 12 hpi with IC_50_ of 4.36 ±1.02 μM (Figure 1F). Quercetin did not exert cytotoxic effect up to 200 μM, and the selectivity index (S.I.) was calculated to be greater than 45 (Table 1). Collectively, these data demonstrated potent anti-RV potential of quercetin at non-cytotoxic concentration.

### Anti-rotaviral role of quercetin is virus strain-independent

**Figure 2:**
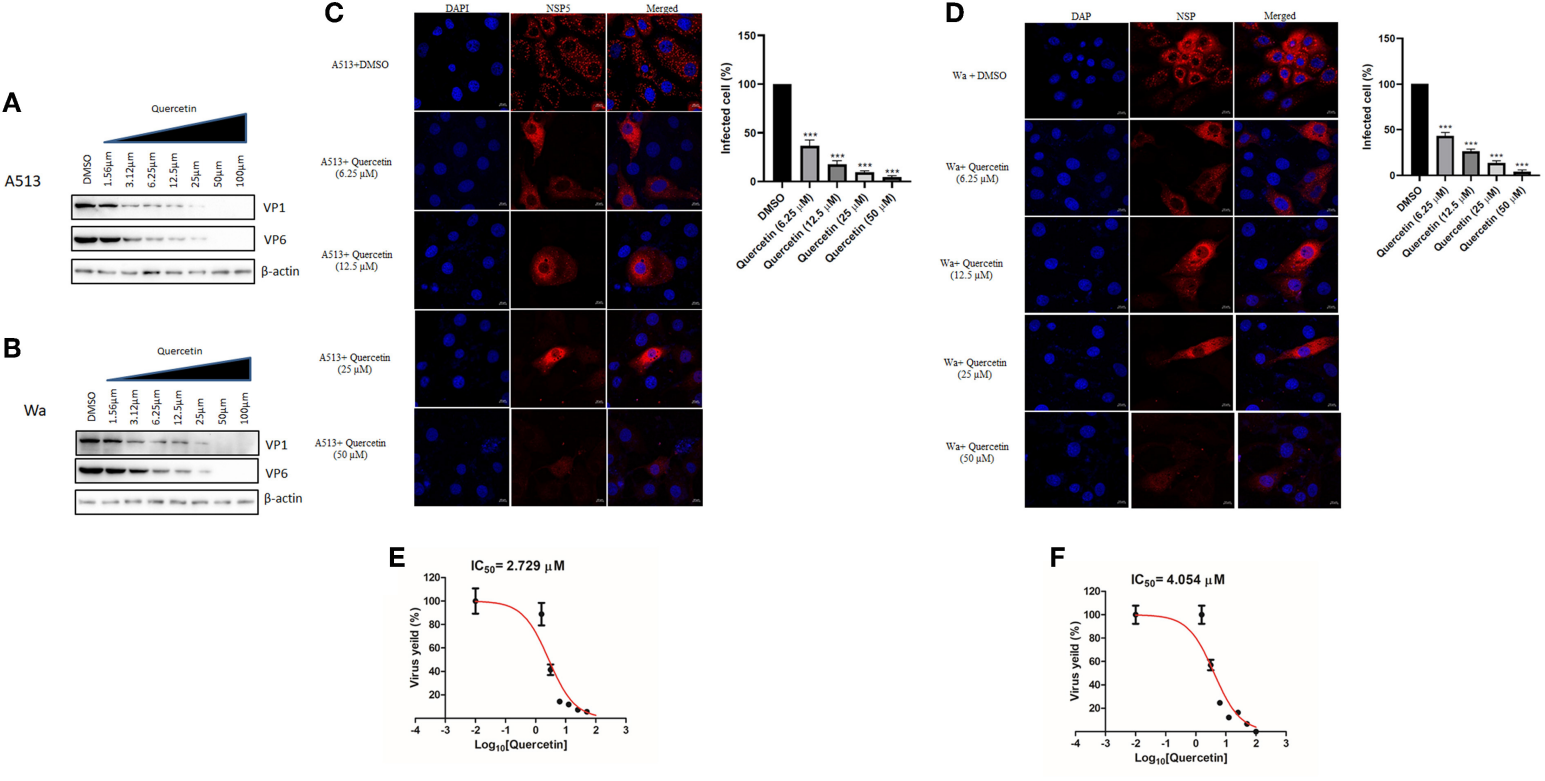
Quercetin exhibits anti-rotaviral activity independent of viral strain: **(A)** Cell lysates were prepared to perform Western blot by infecting MA104 cells with RV-A513 strain followed by treatment with DMSO or increasing concentration of quercetin (1.56, 3.125, 6.25, 12.5, 25, 50, and 100 μM) to assess viral protein expression. The expression of β-actin is checked as internal loading control. **(B)** Similarly, RV-Wa strain-infected MA104 cells are treated with DMSO or increasing doses of quercetin (1.56, 3.125, 6.25, 12.5, 25, 50, and 100 μM) to check viral proteins along with β-actin as loading control. **(C,D)** MA104 cells were infected with **(C)** RV-A5-13 and **(D)** RV-Wa (MOI 3) in the presence of DMSO and quercetin (6.25, 12.5, 25, and 50 μM) for 6 hpi. Cells were fixed, permeabilized, and stained primarily with anti-NSP5 antibody (raised in rabbit). Next, secondary staining was done with rhodamine-labeled anti-rabbit secondary antibody. DAPI was used for mounting. Finally, cells were visualized with confocal microscope (63× oil immersion); scale bar 10 μm. Viroplasm positive cells are quantified and analyzed by taking into account minimum 100 cells selected randomly from each experimental slides. The data were represented as mean-infected cells (%) ± SD of three experimental replicates. **(E,F)** IC_50_ was calculated from the quantification of viral particle produced by plaque assay which was performed from **(E)** A5-13- and **(F)** Wa-infected MA104 cells treated with DMSO and graded concentration of quercetin (1.56, 3.125, 6.25, 12.5, 25, 50, and 100 μM), for 24 hpi. Each bar represents mean ± SD of three independent experiments (Unpaired student's t-test, ***p < 0.001).

To investigate whether quercetin can reduce RV infection in a strain-independent manner, two other RV strains—bovine RV-A5-13 and human RV-Wa—were used to infect MA104 cells in the presence of increasing concentrations of quercetin for 12 hpi, and cell lysates were used to evaluate VP1 and VP6 expression by Western blot. Protein levels of both VP1 and VP6 faced gradual depletion on treatment with increasing concentrations of quercetin in both RV-A5-13- and RV-Wa-infected MA104 cells, demonstrating broad-spectrum antiviral role of quercetin against RV (Figures 2A,B). Comparable to these data, quercetin treatment also diminished the number of viroplasm-positive cells and infectious particle yield in MA104 cells infected with either RV-A5-13 (IC_50_ of 2.79 ± 1.23 μM) or RV-Wa (IC_50_ of 4.05 ± 1.74 μM) compared to DMSO treatment (Figures 2C,D). S.I. of A5-13 and Wa was found to be >73.26 and 49.38, respectively.

### Anti-rotaviral effects of quercetin are independent of interferon pathway

**Figure 3:**
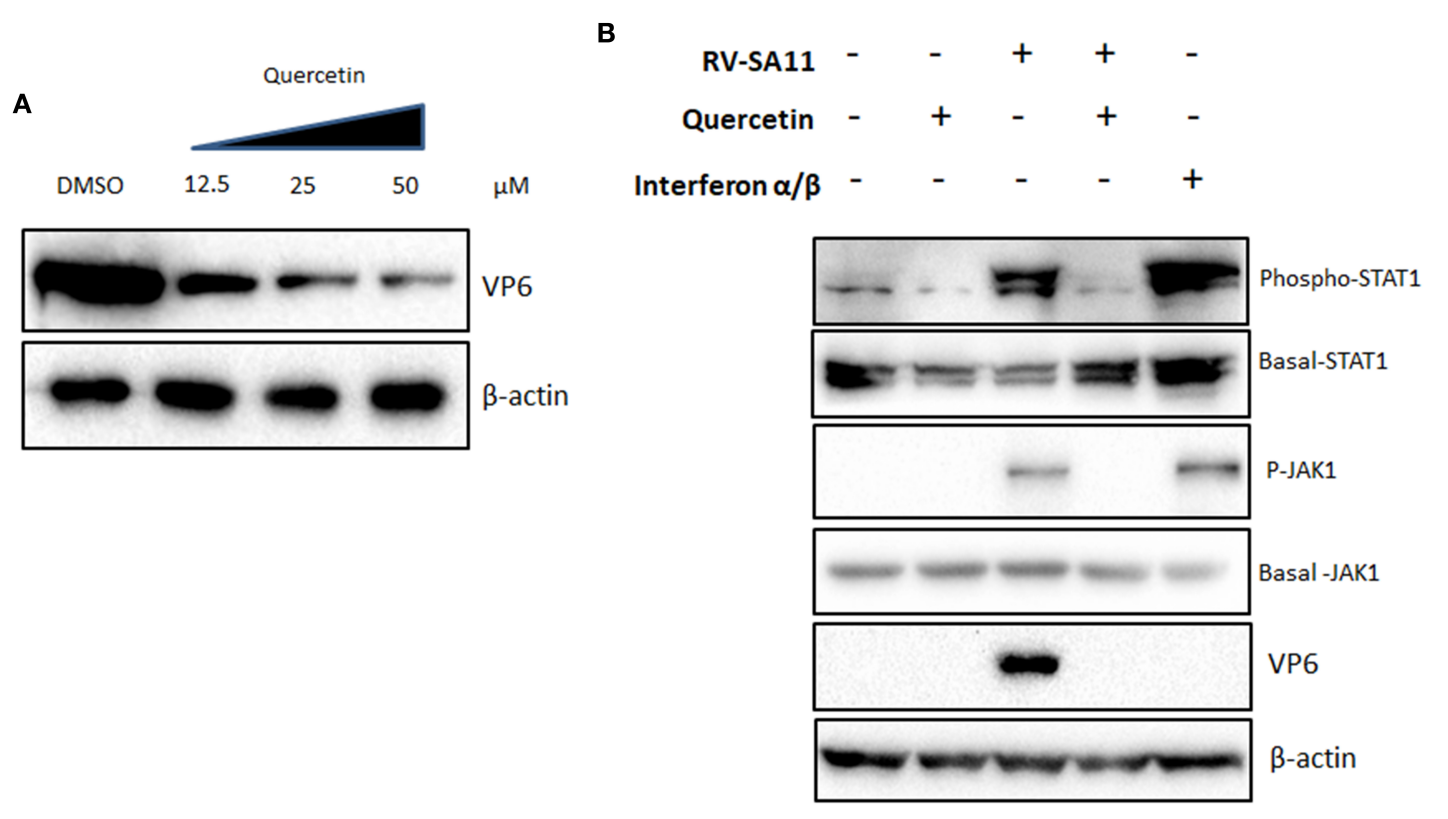
The inhibitory function of quercetin on rotavirus infection is independent of interferon signaling **(A)** Type I IFN signaling mutant vero cells were infected with RV-SA11 (MOI 3) in the presence of increasing concentrations of quercetin (12.5, 25, and 50 μM) for 12 hpi and cell lysates were subjected to Western blot to evaluate the expression of VP6, whereas β-actin was taken as internal loading control. **(B)** The activation of JAK1-STAT1 signaling was checked by Western blot performed from MA104 cell lysates mock-infected or RV-SA11-infected and treated with either DMSO or quercetin (50 μM). IFNα/β conjugate (500 units/ml) was treated as positive control. Protein expression and phosphorylation of JAK1 and STAT1 were assessed along with β-actin as internal loading control.

To investigate the molecular pathway through which quercetin imparts anti-rotaviral potential, first, we checked the ability of quercetin to induce non-specific IFN signaling. To analyze this, interferon-deficient vero cells (type I IFN signaling mutant) were infected with RV-SA11 (MOI 3) in the presence of increasing concentrations of quercetin (12.5, 25, and 50 μM) for 12 hpi, and cell lysates were used to assess the expression of VP6. Consistent with the previous observations, the result showed a gradual decline in the expression of RV-VP6 protein in the presence of increasing doses of quercetin in comparison with DMSO treatment suggesting an interferon-independent role of quercetin in the regulation of RV infection (Figure 3A). To assess whether quercetin can modulate RV-induced IFN pathway, the activation status of Janus-Kinase 1 (JAK1)-Signal transducer and activator of transcription (STAT1) pathway was checked in RV-SA11-infected MA104 cells in the presence and absence of quercetin. MA104 cells treated with IFN-α/β conjugate (500 units/ml) were used as positive control. Quercetin was treated at 50 μM concentration in all following experiments at which the inhibition of rotavirus protein expression has been found to be maximum. Quercetin treatment alone did not induce IFN pathway. Rotavirus-induced phosphorylation of JAK1 and STAT1 reduced significantly in the presence of quercetin (50 μM) compared to DMSO control (Figure 3B). However, there was no direct effect of quercetin on IFN-α/β-induced JAK1/ STAT1 activation (Supplementary Figure S2). These results suggest that anti-rotaviral role of quercetin is not mediated by modulating IFN signaling pathway.

### Quercetin impairs rotavirus infection by inhibiting NF-κB pathway

**Figure 4:**
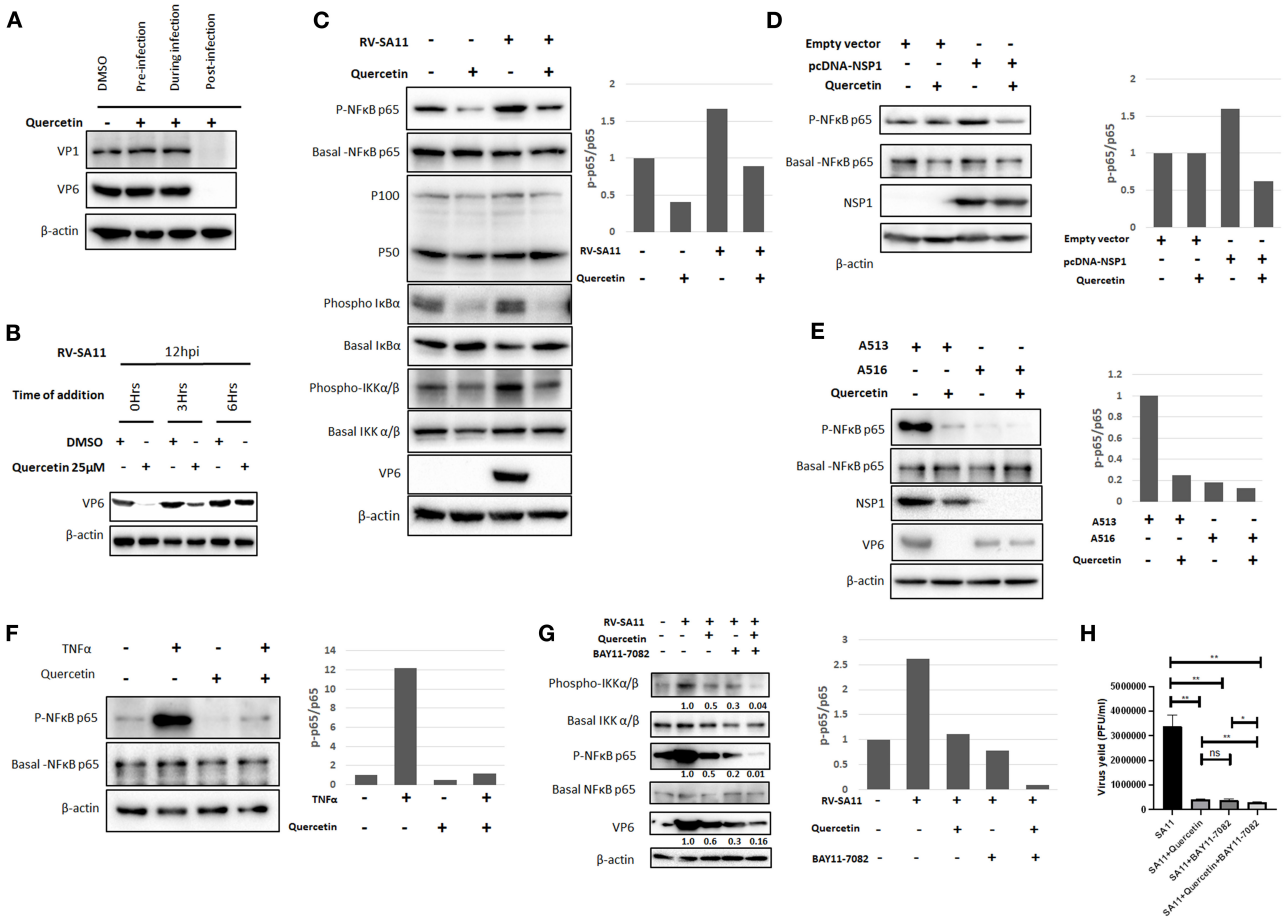
Quercetin restricts rotavirus infection by inhibiting NF-κB pathway. **(A)** RV-SA11 (MOI 3)-infected MA104 cells (12 hpi) were treated with DMSO or quercetin (50 μM) 1 h before adding the virus, during virus addition, and 1 h after addition of the virus to assess the effect of quercetin on viral protein expression on the basis of time of addition. Western blot was performed to check VP1 and VP6 along with internal loading control β-actin. **(B)** SA11 (MOI 3)-infected MA104 cells (12 hpi) were treated with DMSO or quercetin (50 μM) at 0, 3, and 6 hpi, respectively, to assess VP6 expression keeping β-actin as loading control. **(C)** Protein expression and phosphorylation status of chief players of NF-κB signaling pathway such as NF-κB p65, NF-κB p50/p100, IκBα, and IKKα/β were evaluated by SDS-PAGE. Cell lysates were prepared by mock-infected or RV-SA11 (MOI 3)-infected MA104 cells for 6 hpi in the presence of either DMSO or quercetin (50 μM). The expression of VP6 and β-actin served the purpose of infection marker and internal loading control, respectively. The ratio of phospho p65 and basal p65 was plotted in bar graph. **(D)** pcDNA-NSP1 was transfected in MA104 cells to overexpress RV-NSP1 which triggers the activation of NF-κB p65. Next, the transfected cells were either treated with DMSO or quercetin (50 μM). Consequently, the cell lysates are subjected to Western blot to detect the expression of phospho and basal NF-κB p65, NSP1, and internal loading control β-actin. Phospho p65 level was normalized against basal p65 and represented in bar graph. **(E)** MA104 cells are infected RV strain A5-13 (wild-type NSP1) and A5-16 (mutant NSP1), respectively, followed by administration of DMSO or 50 μM quercetin. Cell lysates are run for SDS-PAGE for the detection of phospho and basal NF-κB p65, NSP1, VP6 (infection marker), and β-actin (loading control). The ratio of phospho p65 and basal p65 was represented by bar graph. **(F)** TNFα (10 nM) is administered to MA104 cells to induce NF-κB signaling and also co-treated with quercetin (50 μM) to check the effect of quercetin on TNFα-induced NF-κB pathway. Western blot analysis was done to check expression and phosphorylation of NF-κB p65, and β-actin was checked as internal loading control. Phospho p65 is normalized against basal p65 and plotted in bar graph. **(G)** BAY11-7082 (10 μM) is added to RV-SA11 (MOI 3)-infected MA104 cells (6 hpi) either alone or co-treated with quercetin (10 μM) to check VP6, NF-κB, IKKα/β, and β-actin (loading control) protein levels by Western blot. **(H)** Plaque assay was performed to assess viral titer from SA11-infected MA104 cells treated with DMSO, quercetin (10 Mm), BAY11-7082 (10 μM), and quercetin co-treated with BAY11-7082, respectively. Phospho p65 and basal p65 ratio is represented in bar graph. Each bar represents mean ± SD of three independent experiments (Unpaired student's t-test, ns denotes *p* value not significant, *p < 0.05, **p < 0.01).

To understand whether quercetin affects rotavirus infection at pre-entry, during entry, or post-entry phase of infection cycle, the expression of two viral structural proteins VP1 and VP6 was assessed in RV-SA11-infected MA104 cells (12 hpi) treated with either DMSO or quercetin (50 μM) for 2 h prior to virus adsorption, during adsorption, or throughout infection post-adsorption, respectively (Figure 4A). Both VP1 and VP6 proteins were found to be diminished by quercetin only in cells treated post-infection compared to DMSO-treated cells. The expression of VP1/VP6 was not affected when quercetin was chased at pre-entry or during entry of RV-SA11 suggesting that quercetin inhibits rotavirus infection by modulating viral replication at the post-entry phase. To dissect the post-entry phase of action, time of addition experiment was performed where SA11-infected MA104 cells were treated with either DMSO or quercetin at 0, 3, and 6 hpi and further incubated for 12 hpi followed by the expression analysis of VP6 by Western blot. It was observed that there was no significant inhibitory effect of quercetin if added at 6 hpi on RV replication as assessed by VP6 expression analysis. However, when treated at early hours (0–3 hpi), quercetin showed significant anti-rotaviral effects as demonstrated by reduced VP6 expression. This suggests that quercetin modulates viral replication cycles during early stages (Figure 4B).

We further extended our observations to explore the underlying molecular mechanism by which quercetin imparts its anti-rotaviral action. The previous report showed the inhibitory effect of quercetin on the activation of NF-κB pathway (Granado-Serrano et al., 2010; Chirumbolo, 2013; Indra et al., 2013; Zhang et al., 2015; Cheng et al., 2019). Rotavirus infection has also been reported to activate NF-κB pathway during early hours of infection (Rollo et al., 1999; LaMonica et al., 2001; Holloway et al., 2009; Bagchi et al., 2010). Therefore, we intended to check whether quercetin inhibits rotavirus infection by modulating the activation of NF-κB pathway. To assess this, both the basal level and phosphorylation status of the key components of NF-κB signaling pathway were assessed in MA104 cells either mock-infected or infected with RV-SA11 (MOI 3) in the presence or absence of quercetin (50 μM) at 6 hpi by Western blot (Figure 4C). Consistent with the previous report, phosphorylation of NF-κB p65 was found to be upregulated at 6 hpi compared to mock infection; however, quercetin treatment diminished rotavirus-induced phosphorylation of NF-κB p65 without altering the basal level expression of both p65 and p100/50. The ratio of phospho-p65 and basal p65 was calculated and plotted in bar graph. Similar trend was observed in phospho-IκBα expression as it was found to be reduced following quercetin treatment in both mock-infected and RV-SA11-infected cells compared to DMSO-treated controls. Contrastingly, basal form of IκBα increased in response to quercetin compared to DMSO-treated controls in both mock-infected and RV-SA11-infected cells. We further assessed the phosphorylation status of IKKα/β, the upstream kinase of IκBα. Western blot revealed that the expression of active phosphorylated form of IKKα/β declined significantly upon quercetin treatment in both uninfected and infected cells compared to their respective DMSO controls (Figure 4C). Overall, these data imply that quercetin hinders the activation of NF-κB signaling pathway during early rotavirus infection.

Rotaviral non-structural protein 1 (NSP1) is the viral factor that mediates the activation of NF-κB (Bagchi et al., 2010). Therefore, we further investigated whether quercetin can inhibit NSP1-induced activation of NF-κB pathway. The ectopic expression of RV-NSP1 in MA104 cells induced the phosphorylation of NF-κB which was found to decline in the presence of quercetin, demonstrating the ability of quercetin to suppress NSP1-induced activation of NF-κB (Figure 4D). In addition, we substantiated our observation by assessing effects of quercetin in MA104 cells infected with either A5-13 (encoding wild-type NSP1) or A5-16 (encoding mutant NSP1). Robust phosphorylation of NF-κB p65 was observed in A5-13-infected cellular lysates, whereas the absence of p-NF-κB p65 in A5-16-infected cellular lysates confirmed the inability of NSP1 mutant A5-16 to induce NF-κB activation. Interestingly, in contrast to A5-13-infected cells, antiviral effect of quercetin was not significant in A5-16-infected cells as assessed by the expression of VP6 (Figure 4E). Consistent with this data, we did not observe any significant effect of quercetin in viral particle production from A5-16-infected cells at 24 hpi (Supplementary Figure S3). Overall, the data suggest that the antiviral activity of quercetin is due to the inhibition of NSP1-induced NF-κB activation (Figure 4E). In addition, quercetin also inhibited TNFα (10 nM)-induced activation of NF-κB pathway which further strengthened the specific inhibitory effect of quercetin on NF-κB pathway (Figure 4F). To further confirm that quercetin impedes rotaviral infection by regulating NF-κB pathway, RV-infected cells were treated with either BAY11-7082 (10 μM), a specific inhibitor of IKK alone or in combination with quercetin (10 μM). Interestingly, the result showed comparable reduction in viral protein VP6 level to that of phospho-NF-κB p65 level when treated with quercetin, BAY11-7082, and both together in the RV-SA11-infected MA104 cells (Figure 4G). The quantification of viral titer at 12 hpi revealed that there was no significant difference in reduction of virus particles in RV-SA11-infected MA104 cells treated with quercetin alone (7.97-fold reduction), BAY11-7082 alone (8.62-fold reduction), or with both (11.82-fold reduction) compared DMSO-treated RV-SA11-infected cells (Figure 4H). This confirmed that anti-rotaviral effects of quercetin were due to the inhibition of RV-induced NF-κB pathway as in the presence of BAY11-7082, no additive or synergistic antiviral effects were observed.

### Quercetin represses rotavirus infection *in vivo*

**Figure 5:**
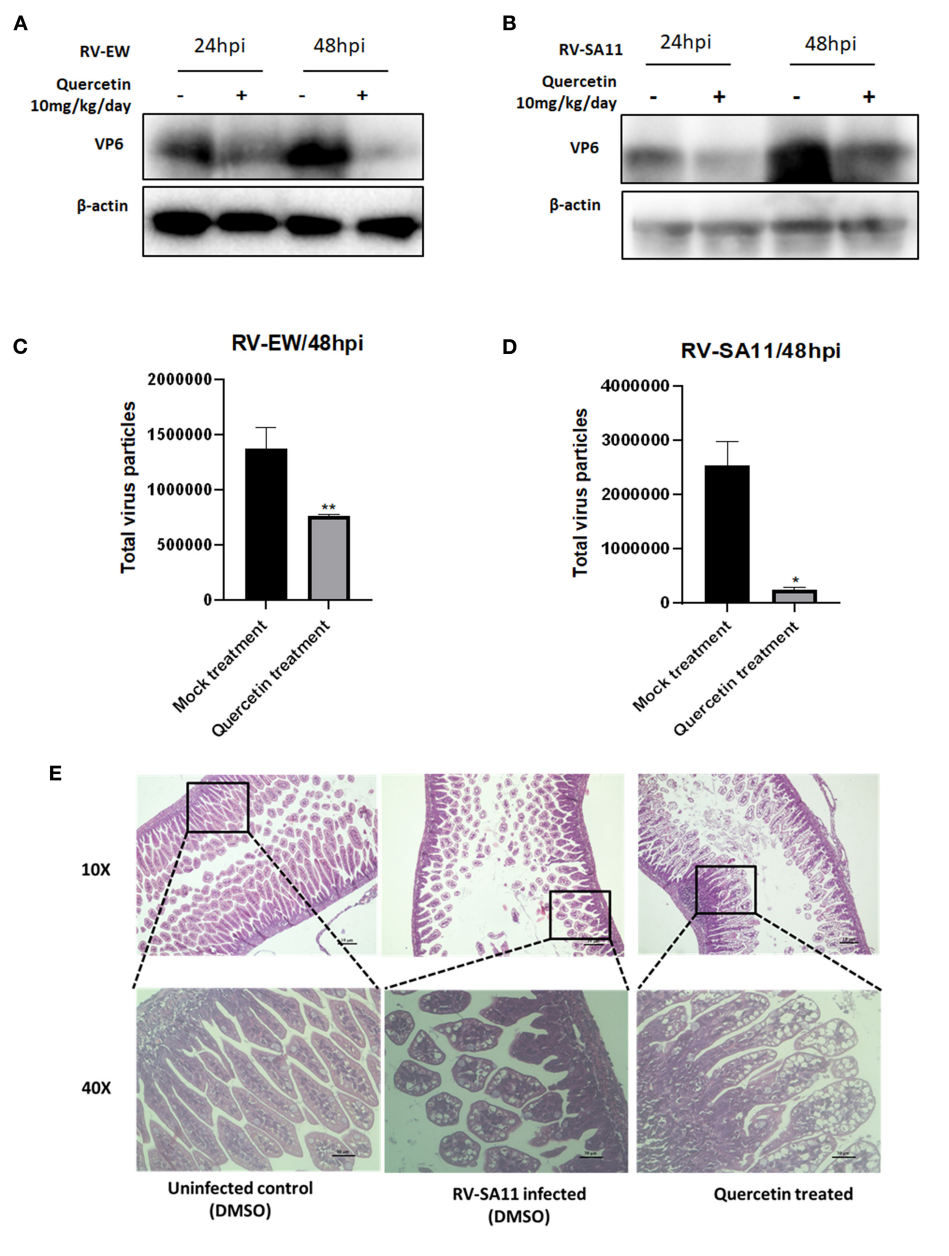
Quercetin hinders rotavirus infection in mice model. **(A,B)** Expressions of VP6 and β-actin proteins were assessed by Western blot performed from the small intestinal tissue of 4-day-old suckling mice infected with **(A)** rotavirus murine strain Ew and **(B)** rotavirus simian strain SA11 followed by quercetin treatment after 24 and 48 h. **(C,D)** Stool samples were collected from suckling mice infected with RV and treated either with DMSO or quercetin for 48 h. Total virus particles were quantified afterward. Each bar represented mean ± SD of three independent experiments (Unpaired student's t-test, *p < 0.05, **p < 0.01). **(E)** HE staining of small intestinal tissue section of 5-day-old mice either uninfected or infected with RV-SA11 in the presence or absence of quercetin (10 mg/kg/day). Scale bars are 10 μm and 30 μm.

Lastly, anti-rotaviral activity of quercetin was assessed in mice model. Five-day-old BALB/c suckling mice were infected with either murine rotavirus strain EW (10^7^ PFU) or simian RV strain SA11 (10^7^ PFU) through oral gavages. After 12 h of RV inoculation, suckling mice were treated with 10 mg/kg/day quercetin along with DMSO vehicle for up to 1 day (24 h) and 2 days (48 h). Incubated mice were euthanized, and small intestine was isolated to prepare intestinal tissue homogenate. Viral protein expression in response to quercetin administration was examined by Western blot. The expression of VP6 protein was found to be significantly reduced in quercetin-treated RV (EW and SA11)-infected mice at both 1-day and 2-day post-infection compared to DMSO-treated control (Figures 5A,B). Total fecal samples were collected from two groups of RV-infected mice, mock (DMSO)-treated mice, and quercetin (10 mg/kg/day)-treated, respectively, for quantifying rotavirus particles. The results disclosed a decrease in virus particle shedding in stools of quercetin-treated mice compared to untreated control mice (Figures 5C,D). Consistent with the previous results, quercetin treatment also restored the normal morphology of the small intestinal villi which has been damaged by RV-SA11 infection (Figure 5E). The large intestinal view of the suckling mice showed quercetin treatment has reformed solid stool as opposed to watery diarrhea observed in RV-infected untreated mice (Supplementary Figure S4).

## Discussion

Acute viral gastroenteritis in children is a major cause of childhood mortality in resource-poor settings. Thus, continuous efforts are being made to develop and introduce vaccines against diarrheagenic pathogens worldwide. However, for viral gastroenteritis in addition to vaccine, there is a need to develop antivirals to reduce the morbidity and duration of illness. As synthetic antiviral drugs cause adverse side effects affecting human health in an indirect manner, attention has been shifted toward plant-based treatments against viral infection due to reduced toxicity. Furthermore, plant metabolites and bioactive compounds having plethora of multi-dimensional properties can supersede the difficulty related to drug resistance (Ghildiyal et al., 2020). Quercetin, an FDA-approved dietary bioflavonoid polyphenolic compound obtained from diverse food sources including fruits, vegetables, and leaves, has immense antioxidant and anti-inflammatory properties (Thangasamy et al., 2009; Dewi et al., 2020; Mehrbod et al., 2020; Brito et al., 2021). Antiviral effect of quercetin is also reported in various other viral infections such as influenza, dengue, Ebola, and rhinovirus (Ganesan et al., 2012; Wu et al., 2015; Dewi et al., 2020; Fanunza et al., 2020; Mehrbod et al., 2020).

This study elucidated the robust anti-rotaviral potential of quercetin, a plant flavonoid *in vitro* and *in vivo*. In contrast to a single report on mild anti-rotaviral activity of quercetin (Bae et al., 2000), we observed strain-independent and dose-dependent potent anti-rotaviral effects of quercetin both *in vitro* and *in vivo* as observed by RV protein expression and infectious viral particle production (Figures 1, 2, 5). The IC_50_ values ranged from 2.79 to 4.36 μM (Figure 1F), and the calculated S.I. index for anti-rotaviral activity is greater than 45.87 in case of SA11 strain (Supplementary Figure S1). Quercetin being a natural plant derivative has shown no cytotoxic effect up to a concentration of 200 μM. This suggests that it can be safely used in human. Quercetin treatment regenerated the damaged small intestinal villi of RV-infected suckling mice and restored solid stool formation in treated mice when compared to RV-infected control mice which had watery diarrhea.

Quercetin has previously been reported to differentially regulate interferon (IFN) signaling in peripheral blood mononuclear cells (Nair et al., 2002). However, we did not observe induction of IFN signaling in quercetin-treated mock-infected or RV-infected cells. In cells treated with quercetin, reduced activation of RV-induced IFN signaling was observed probably due to low virus replication (Figure 3).

Quercetin is reported to exert its antiviral effect via alteration of an array of host pathways including ROS signaling, TNF-α, autophagy, the expression of cytokines, and also via directly targeting viral proteins including NA of H1N1, RNA polymerase of influenza A and B, and catalytic pocket of SARS-CoV 3CLpro (Pisonero-Vaquero et al., 2014; Gansukh et al., 2016; Liu et al., 2016; Lee et al., 2017; Granato et al., 2019; Jo et al., 2020). RV infection also results in the activation of various host signaling pathways including PI3K, Akt, ERK, JNK, p38, and NF-κB which have been shown to be modulated by quercetin (Holloway et al., 2009; Bagchi et al., 2010; Soliman et al., 2018). Notably, we did not see any significant effect on PI3K, Akt, ERK, JNK, and p38 signaling in RV-infected quercetin-treated cells (data not shown). However, we observed that anti-RV effect of quercetin involves the inhibition of pro-survival and inflammatory signaling related to NF-κB signaling pathway. This is concurrent with previous reports of quercetin-mediated inhibition NF-κB signaling in several disease models like cancer and inflammation (Granado-Serrano et al., 2010; Chirumbolo, 2013; Indra et al., 2013; Zhang et al., 2015; Cheng et al., 2019).

The activation of NF-κB signaling by RV-NSP1 during early hours of RV infection (SA11, RRV, Wa, and A5-13) has been reported in many studies (Rollo et al., 1999; LaMonica et al., 2001; Holloway et al., 2009; Bagchi et al., 2010). In contrast, Graff et al. (2009) reported the inhibition of NF-κB as a result of β-TrCP degradation mediated by NSP1 in RV-OSU- and RV-NCDV-infected cells (Graff et al., 2009). These contrasting findings might be due to structural differences between NSP1 proteins of different RV strains (Morelli et al., 2015). As there are contradictory reports regarding the activation of NF-κB pathway during RV infection, we independently confirmed the activation of NF-κB pathway at 6 hpi of RV-SA11 infection in MA104 cells in this study. Subsequently, we found that the administration of quercetin reduces RV induced elevation of phospho-NF-κB p65, p-IκBα, and p-IKK during early hours (2–8hpi) of infection (Figure 4C). Consistent with the previous studies (Ruiz et al., 2007; Chen et al., 2020), we also observed downregulation of TNFα-induced NF-κB activation following quercetin treatment (Figure 4F). A family of inhibitory proteins IκB holds back NF-κB proteins in the cytoplasm in an inactive form. The canonical NF-κB pathway is initiated when a multi-subunit IκB kinase (IKK) complex phosphorylates IκBα which triggers its ubiquitin-dependent proteasomal degradation. As a result, NF-κB p65 becomes phosphorylated and activated, and free phospho-NF-κB p65 undergoes rapid nuclear translocation (Liu et al., 2017). The diminished level of IKK, IκBα and phospho-NF-κB p65 level in quercetin-treated cells, depicts the inhibition of IKK which cannot phosphorylate and degrade IκBα, subsequently stabilizing NF-κB at cytosol in the inactive state. Thus, quercetin modulates the IKK complex activation during RV infection. This is consistent with the previous study which showed that quercetin suppresses NF-κB pathway by potently inhibiting IKKα and IKKβ (Peet and Li, 1999). To understand whether the inhibition of NF-κB by quercetin through IKK complex is responsible for reduction in rotaviral infection, BAY11-7082, a specific inhibitor of IKK, is used (Figure 4G). Surprisingly, the data demonstrated proportional reduction of phospho-NF-κB to the level of decrease in VP6 level when RV-SA11-infected cells were treated with quercetin, BAY11-7082 alone, and co-treated with quercetin. Thus, quercetin plausibly functions as anti-rotaviral compound by inhibiting IKK/NF-κB axis by inhibiting phosphorylation and activation of IKK complex as a result abrogates NF-κB activation. However, the mechanism, by which quercetin inhibits phosphorylation of IKK complex, further needs to be evaluated.

Furthermore, rotavirus non-structural protein 1 (NSP1) is reported to hinder apoptosis for propagation of viral progeny by stimulating NF-κB cell survival pathway to favor propagation of viral progeny (Bagchi et al., 2010). Consistent with the infection scenario, NSP1 transfection amplified phospho-NF-κB p65 levels which were downregulated by quercetin (Figure 4D). Quercetin treatment could not exert its antiviral effects on NSP1 mutant strain A5-16 infection when compared to wild-type A5-13 strain as due to the lack of functional NSP1 in A5-16, there is no induction of phospho-NF-κB p65 (Figure 4E). Unlike other viruses like influenza where quercetin imparts antiviral activity at virus–host cell fusion step (Wu et al., 2015) or Ebola virus where quercetin inhibits anti-IFN activity of EBOV VP24 (Fanunza et al., 2020), in context of rotavirus infection, quercetin utilizes a different strategies to exert its antiviral effect.

In summary, the study highlights the anti-rotaviral potential of quercetin both *in vitro* and *in vivo*. Being a plant-based natural compound, it has minimal cytotoxicity and can be applied for successful disease management during RV infection combined with oral rehydration therapy to help minimize mortality and morbidity in infants.

## Data availability statement

The raw data supporting the conclusions of this article will be made available by the authors, without undue reservation.

## Ethics statement

The animal study was reviewed and approved by Institutional Animal Ethical Committee (IAEC), ICMR-National Institute of Cholera and Enteric Diseases.

## Author contributions

MC-S and SB conceived the study. SB, RS, AM, and MC-S designed the experiments and contributed to drafting and revising the manuscript. SB, RS, and PH performed the experiments. PH and HK contributed to animal experiments. KK and S-iM contributed valuable scientific inputs. All authors read and approved the final manuscript.

## Funding

This work was supported partly by Okayama University Project through the Japan Agency for Medical Research and Development (AMED; Grant No. JP21wm0125004).

## Conflict of interest

The authors declare that the research was conducted in the absence of any commercial or financial relationships that could be construed as a potential conflict of interest.

## Publisher's note

All claims expressed in this article are solely those of the authors and do not necessarily represent those of their affiliated organizations, or those of the publisher, the editors and the reviewers. Any product that may be evaluated in this article, or claim that may be made by its manufacturer, is not guaranteed or endorsed by the publisher.
