# Supplementary material for: Quercetin, a flavonoid, combats rotavirus infection by deactivating rotavirus-induced pro-survival NF-κB pathway
Source: Front Microbiol. 2022 Aug 2;13:951716. doi: 10.3389/fmicb.2022.951716 (PMC9379144; doi:10.3389/fmicb.2022.951716)
Supplement: Supplementary file 1 [file Data_Sheet_1.docx]

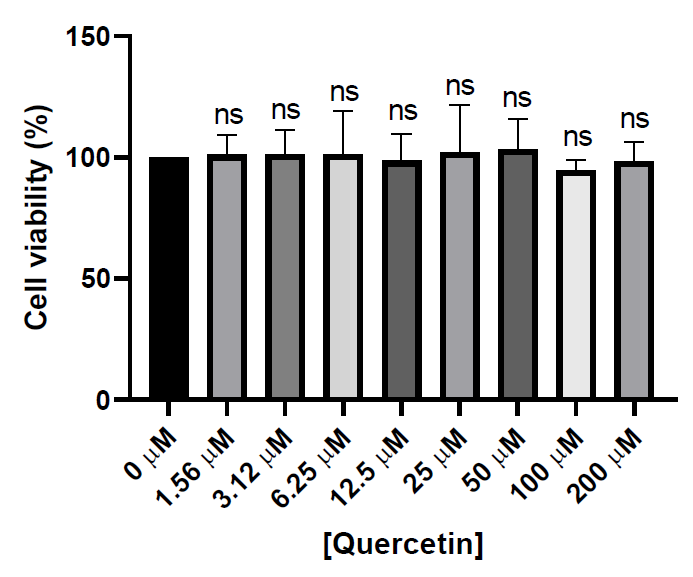


**Fig. S1** Effect of quercetin on cell viability. MA104 cells were treated with indicated concentrations of quercetin for 48 hours and cell viability was measured by MTT assay.

Phospho-STAT1

Interferon α/β

- - + +

- + - +

Quercetin


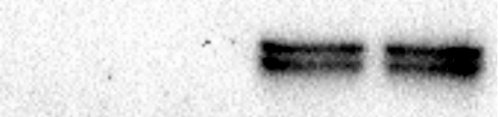

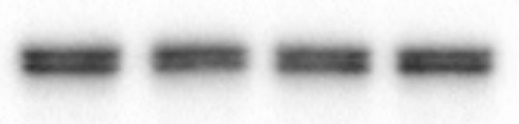


Basal-STAT1


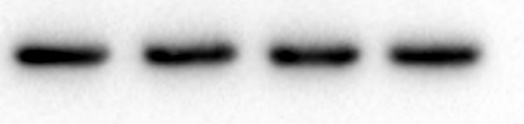


β-actin


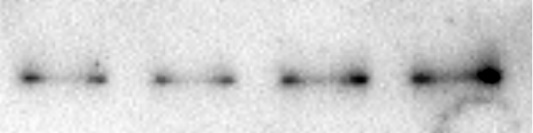

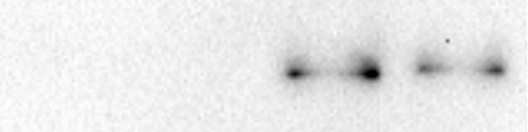


P-JAK1

Basal -JAK1

**Fig. S2** Quercetin at 50μM dose was treated to MA104 cells along with IFNα/β conjugate (500units/ml) to evaluate effect of quercetin on IFN induced JAK1-STAT1 signaling. JAK1 and STAT1 phosphorylation and protein expression were checked keeping β-actin as internal loading control.

**Fig. S3** Production of infectious virus particles from MA104 cells infected with either A5-13 or A5-16 both in presence and absence of quercetin (50 μM) for 24 hpi was assessed by plaque assay. The data is represented as log_10_ (PFU/ml). Each bar represented mean ± SD of three independent experiments (Unpaired student’s t-test, *p< 0.05, **p<0.01).


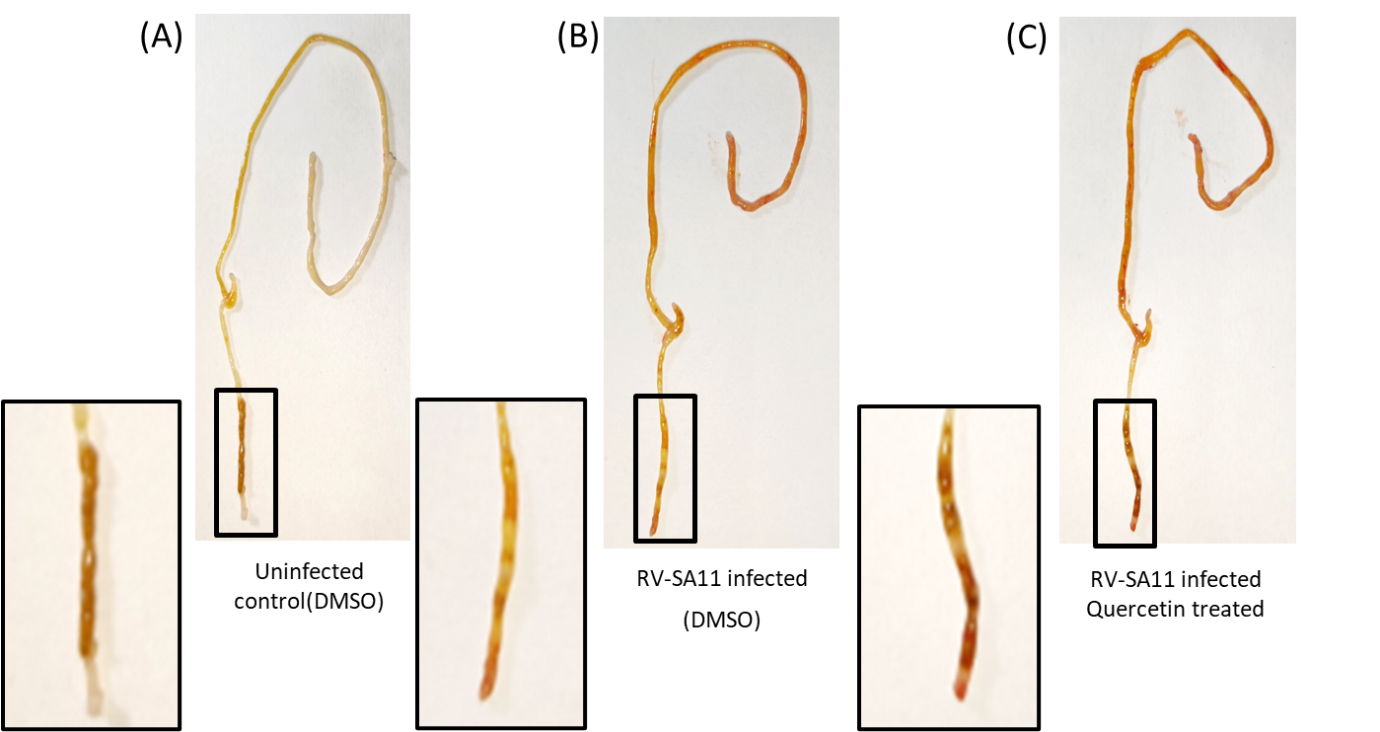


**Fig. S4** Visualization of large intestine of RV-SA11-infected suckling mice either uninfected or infected with RV-SA11 in the presence of DMSO or quercetin (10 mg/kg/day). Data revealed decreased accumulation of watery diarrhea in the large intestine of drug treated mice compared to that of untreated infected mice.
